# Geschlechtsspezifische Unterschiede spezieller Tumorerkrankungen

**DOI:** 10.1007/s00108-023-01551-9

**Published:** 2023-07-17

**Authors:** Nathalie Lehnen, Michael Hallek

**Affiliations:** Klinik I für Innere Medizin, Universitätsklinikum Köln (AöR), Kerpener Str. 62, 50937 Köln, Deutschland

**Keywords:** Gendermedizin, Therapieassoziierte Toxizität, Tumorerkrankungen/geschlechtsspezifische Risikofaktoren, Tumorerkrankungen/geschlechtsspezifisches Outcome, Präzisionsmedizin, Gender medicine, Treatment-associated toxicity, Neoplasms/sex-specific risk factors, Neoplasms/sex-specific outcome, Precision medicine

## Abstract

**Hintergrund:**

Zahlreiche Daten zeigen, dass Geschlecht und Gender als relevante Modulatoren bestimmter onkologischer und hämatologischer Erkrankungen einen zunehmenden Stellenwert in der Präzisionsmedizin verdient haben. Dieser Beitrag soll eine Zusammenfassung des aktuellen Wissensstands über Geschlechtsunterschiede in Inzidenz und Outcome spezieller Tumorerkrankungen bieten und mögliche zugrunde liegende Ursachen näher beleuchten.

**Material und Methoden:**

Auswertung und Diskussion von Grundlagenarbeiten, Metaanalysen und klinischen Studien

**Ergebnisse:**

Es bestehen für eine Vielzahl der onkologischen Erkrankungen signifikante geschlechtsspezifische Unterschiede in Inzidenz, Ansprechraten und Mortalität. Zumeist haben Männer ein schlechteres Outcome, während Frauen höhere therapieassoziierte Toxizität und distinkte Präsentationen in jungem Alter aufweisen. Hormonelle, immunologische und pharmakologische Ursachen liegen nahe.

**Schlussfolgerung:**

Eine fortschrittliche in Bezug auf die einzelnen Patient*innen individualisierte Therapie in Onkologie und Hämatologie wird sich zukünftig daran messen, die bestehenden relevanten Unterschiede zwischen den Geschlechtern im klinischen Alltag zu berücksichtigen und in Studien zugrunde liegende Mechanismen weiter zu beleuchten, um eine bestmögliche Behandlung für onkologische Patient*innen zu garantieren und zukünftig zu optimieren.

## Abstract (en)

**Background:**

Numerous data show that sex and gender have gained increasing importance in precision medicine as relevant modulators of specific oncological and hematological diseases. The purpose of this article is to provide a summary of the current state of knowledge on sex differences in the incidence and outcome of specific malignancies and to further elucidate possible underlying causes.

**Material and methods:**

Evaluation and discussion of basic research studies, meta-analyses, and clinical trials.

**Results:**

There are significant sex-specific differences in the incidence, response rates, and mortality for a variety of oncological diseases. For the most part, men have poorer outcomes, whereas women have higher treatment-associated toxicities and distinct presentations at younger ages. Hormonal, immunological, and pharmacological causes are suspected.

**Conclusion:**

Advanced patient-individualized treatment in oncology and hematology will be measured in the future by the implementation of the existing relevant sex differences in the clinical practice and further investigations on underlying mechanisms in studies in order to guarantee and to optimize the best possible treatment for oncological patients in the future.

## „Sex matters“ – auch in der Onkologie

Geschlechtsspezifische Unterschiede als Einflussfaktoren in Bezug auf Gesundheit, Risikofaktoren, therapeutische Maßnahmen und deren Outcome wurden in den letzten Jahren mit zunehmendem Interesse beforscht und mit wachsender Relevanz für klinische Entscheidungen bewertet [1, 2]. Während in der Onkologie und Hämatologie das klinische Handeln und Forschungsinhalte häufig noch geschlechts- und genderblind bleiben, besteht in Fächern wie der Kardiologie und Pharmakologie bereits ein größeres Bewusstsein für eine geschlechtssensible Patientenbehandlung im klinischen Alltag und Studiendesign [3–6]. Dabei liegen viele wegweisende Daten zu geschlechtsspezifischen Unterschieden vor, unter anderem in Toxizitäts- und Ansprechraten verschiedener Immun- und Chemotherapeutika mit Einfluss auf das Outcome onkologischer Patient*innen. Diese Unterschiede sollen im Folgenden näher beleuchtet werden.

In der Onkologie bleiben klinische Praxis und Forschung bislang häufig noch geschlechts- und genderblind

Bereits die reine Betrachtung allgemeiner epidemiologischer Daten macht schnell klar, dass wir in der Onkologie deutlichen Unterschieden zwischen den Geschlechtern gegenüberstehen. So findet sich bei der überwiegenden Mehrzahl der (nicht geschlechtsbezogenen) Krebserkrankungen ein erhöhter Männeranteil in Inzidenz und Mortalität [7]. Die größten geschlechtsbezogenen Unterschiede im Outcome lassen sich für Karzinome von Larynx, Ösophagus, Blase und Lunge verzeichnen [8, 9]. Lange Zeit wurden unterschiedliche Expositionen gegenüber Umweltfaktoren (Asbest) und Noxen (Alkohol, Drogen, Tabakkonsum) sowie ein genderspezifisches Risikoverhalten als eine hinreichende Erklärung hierfür angenommen – ein Statement, das unter Betrachtung aktueller Daten zunehmend differenziert werden muss. Für eine Vielzahl der Krebserkrankungen zeigt sich auch nach Bereinigung um bestehende Kovariablen (Risikoverhalten und Karzinogenbelastung) ein erhöhtes relatives Risiko für Männer [8]. Dem gegenüber stehen unter anderem geschlechtsspezifische Unterschiede in der Ansprechrate und Verträglichkeit bestimmter Immuntherapien und Chemotherapeutika mit häufig höherer Toxizität für Patientinnen [10, 11] sowie signifikant unterschiedliche Ansprechraten etablierter sowie neuer Therapien [12].

In der Vergangenheit wurden Daten zu geschlechtsspezifischen Unterschieden in der Inzidenz und Prognose verschiedener Tumorerkrankungen hauptsächlich in epidemiologischen Analysen dargelegt. Untersuchungen der zugrunde liegenden biologischen Aspekte, die subgruppenadjustierte Analysen zum tatsächlichen Einfluss des Geschlechts auf die Prognose ermöglichen könnten, fehlen. Heute sind geschlechtsspezifische, biologische Unterschiede auf hormoneller, immunologischer und molekularer Ebene, die Einfluss auf Karzinogenese, Ansprechraten und Gesamtüberleben („overall survival“ [OS]) nehmen, zunehmend Gegenstand vielversprechender Untersuchungen. Im Folgenden wollen wir geschlechtsspezifische Unterschiede am Beispiel ausgewählter Erkrankungen ausschnittsweise näher beleuchten und mögliche zugrunde liegende Mechanismen näher betrachten.

## Geschlechtsspezifische Eigenschaften des Immunsystems und Hormonhaushalts mit Einfluss auf Tumorgenese und Outcome

Das angeborene und das adaptive Immunsystem weisen grundsätzliche geschlechtsspezifische Unterschiede auf. Neben genetischen Faktoren wie einer fehlenden X‑chromosomalen Inaktivierung und genetischen Polymorphismen nehmen das Alter, der Reproduktionsstatus und die damit verbundenen Sexualhormone geschlechtsabhängigen Einfluss auf das Immunsystem [13]. Unterschiede lassen sich bereits auf Keimbahnebene feststellen. So zeigt sich beispielsweise, erklärt durch eine fehlende X‑chromosomale Inaktivierung, eine höhere Expression des Toll-like-Rezeptors 7 (TLR7) bei Frauen und eine damit einhergehende höhere Produktion von Interferon‑α (IFN-α) nach In-vitro-Stimulation mit TLR7-Liganden [14]. Auf Transkriptionsebene weisen mononukleäre Zellen des peripheren Bluts isoliert aus weiblichen Probandinnen nach Infektionen oder Impfungen eine stärkere Hochregulierung der Expression von proinflammatorischen Genen auf (beispielsweise von „myeloid differentiation primary response protein 88“ [MyD88], Januskinase 2 [JAK2] und Tumor-Nekrose-Faktor [TNF]), insbesondere entlang der TLR-Achse [15]. Zudem zeigen sich deutliche Unterschiede in der Zusammensetzung der Immunzellen. Frauen weisen eine höhere Anzahl an CD4-positiven T‑Zellen sowie ein höheres CD4/CD8-Verhältnis auf [16]. Darüber hinaus zeigen In-vitro-Experimente nach Immunstimulation eine stärkere T‑Zell-Antwort bei Frauen. Unter anderem kann hier eine höhere Anzahl an aktivierten CD4-positiven, CD8-positiven und proliferierenden T‑Zellen bei Frauen im Vergleich zu männlichen Probanden gefunden werden [16]. Darüber hinaus haben Frauen höhere Baseline-Level von Immunglobulinen und B‑Zellen [17]. Insgesamt haben Frauen damit eine stärkere Immunantwort sowohl des angeborenen als auch des adaptiven Systems auf Fremd- und Eigenantigene [13]. Hieraus leiten sich auch erhöhte Inzidenzen von Autoimmun- und Hyperinflammationserkrankungen unter Frauen ab [18].

Bei zunehmender Bedeutung der Immuntherapien im Bereich der Onkologie und Hämatologie ergeben sich hieraus zudem mögliche Erklärungsansätze für geschlechtsspezifische Unterschiede in Ansprechraten und Prognose, wobei detaillierte, die Aspekte verbindende Studien hier bislang fehlen. Metaanalysen klinischer Studien bei unterschiedlichen Tumorerkrankungen legen nahe, dass junge Frauen schlechtere Ansprechraten auf Checkpointinhibitoren aufweisen [19–21]. Castro et al. [22] vertreten die Hypothese, dass junge Frauen stärkere Reaktionen des Immunsystems aufweisen und somit Tumoren, die sich in diesem Lebensabschnitt entwickeln, sich tendenziell „unsichtbarer“ für das Immunsystem verhalten müssen und dadurch weniger Angriffsfläche für Immuncheckpointinhibitoren bieten. Das Gegenteil wird für ältere Männer postuliert [22]. Über verschiedene Krebskohorten hinweg konnte gezeigt werden, dass bei weiblichen und jüngeren Patient*innen Treibermutationen seltener durch Major-histocompatibility-complex(MHC)-I- und MHC-II-Moleküle präsentiert werden, was auf eine stärkere Immunselektion zu Beginn der Tumorgenese hindeutet [22].

Das Ausmaß der Immunselektion in Tumoren variiert mit Geschlecht und Alter

Ein weiterer Bereich von großer geschlechtssensibler Bedeutung in Immunologie und Tumorgenese sind hormonelle Einflüsse. So weist Progesteron eine antiinflammatorische Wirkung auf, Androgene nehmen einen tendenziell immunsupprimierenden Einfluss, während Östrogene, wie Östradiol, eine Verstärkung bestimmter Immunreaktionen bewirken [23]. Östrogene, als wichtige Immunmodulatoren, nehmen darüber hinaus Einfluss auf die Zellteilung, Apoptose und Differenzierung hämatopoetischer Zellen [24]. Ihre Rezeptoren (Östrogenrezeptoren [ER] α und β) werden unter anderem auf B‑ und T‑Lymphozyten, hämatopoetischen Stammzellen [25], Leukämie- und Lymphomzellen [26] sowie auf gesunden als auch Tumorzellen des Ösophagus, Magens, Kolons und der Blase exprimiert [27–29]. Der Einfluss der Geschlechtshormone auf das Immunsystem unterliegt erwartungsgemäß Schwankungen zwischen den Entwicklungsphasen der Pubertät und Menopause [13]. Parallel hierzu wird in vielen Studien und in Bezug auf verschiedene Tumorentitäten ein protektiver Einfluss von Östrogenen auf die Entwicklung verschiedener onkologischer Erkrankungen diskutiert [30].

Östrogene weisen in der Tumorgenese bestimmter Krebsentitäten einen protektiven Einfluss auf

Während ihr Einfluss auf die Tumorgenese innerhalb der Geschlechtsorgane (Mammae, Ovarien, Prostata und Hoden) vielseitig untersucht und therapeutisch adressiert wird [31], liegen teils nur begrenzte Daten zu genauen Effekten auf die Tumorgenese in den geschlechtsunabhängigen Organen vor. Frauen scheinen vor der Entwicklung bestimmter Krebserkrankungen bis zum Erreichen des Klimakteriums besser geschützt zu sein, häufig gleichen sich die Inzidenzraten zwischen Männern und Frauen hiernach zunehmend an [32]. Der Magen-Darm-Trakt gilt als besonders sensibel für Östrogene, sowohl in seiner natürlichen Funktion [33] als auch in Bezug auf die Tumorgenese (siehe Abschnitt „Gastrointestinale Tumoren“). Passend hierzu führt auch in Mausmodellen für Lymphome die Aktivierung des ER‑β zu einer Hemmung von Lymphomwachstum, Vaskularisation und Ausbreitung [34]. Auf der anderen Seite können erhöhte Testosteronspiegel das Zellwachstum fördern und werden insgesamt mit einem gesteigerten Risiko für maligne Melanome und Prostatakarzinome bei Männern [35, 36] sowie für Brust- und Endometriumkarzinome bei Frauen assoziiert [36]. Insgesamt liegen somit diverse Daten vor, die einen starken hormonellen Einfluss auf die geschlechtsspezifische Tumorgenese auch in Organen fern des Reproduktionstrakts zeigen. Klinische Implikationen für präventive und therapeutische Strategien konnten bislang jedoch nicht abgeleitet werden. Tiefer gehende Untersuchungen der diversen hormonellen Einflüsse auf die Tumorgenese und die Frage, ob sich hieraus mögliche Therapie-Targets eröffnen, sind somit von großem Interesse.

## Pharmakologie und Toxizität

Gerade im Fachgebiet der Onkologie mit den relevanten Nebenwirkungen der intensiven und teils stark belastenden Chemo- und Immuntherapien spielen patientenindividuelle Verstoffwechselung, Wirkspiegel und Toxizitätsprofile eine bedeutende Rolle. Pharmakokinetik und Pharmakodynamik unterscheiden sich teils immens zwischen den Geschlechtern. Unter anderem hat der im Mittel 10 % höhere Körperfettanteil bei Frauen Einfluss auf die Wirkstoffverteilung im Körper sowie auf den Wirkeintritt [37]. Eine große Charakterisierung auf molekularer Ebene konnte zeigen, dass 53 % (60/114) der klinisch relevanten (Target‑)Gene eine geschlechtsspezifische Signatur aufweisen [38], was bislang nur unzulänglich in klinischen Studien adressiert wurde. Zudem bestehen Unterschiede im Expressions- und Aktivitätslevel bestimmter arzneimittelmetabolisierender Enzyme, wie Cytochrom P_450_ 3A4 (CYP3A4; erhöhte Aktivität bei Frauen [39, 40]) oder des *MDR1*-Gens (höhere Expression bei Männern), die zu geschlechtsspezifischen Unterschieden von Medikamentenspiegeln beitragen. Für viele Zytostatika besteht eine stark dosisabhängige Ansprech- und Toxizitätsrate, sodass Geschlechterunterschiede in Verteilung und Verstoffwechselung der Medikamente von hoher klinischer Relevanz sind [10].

Bei Frauen sind 5‑FU- und anthrazyklinhaltige Chemotherapieregime mit höherer Toxizität assoziiert

5‑Fluoruracil (5-FU), das als integrativer Bestandteil verschiedener Chemotherapieregime in der Standardbehandlung fortgeschrittener kolorektaler Karzinome, Magenkarzinome und Mammakarzinome eingesetzt wird, zeigte in einer prospektiven pharmakologischen Studie nach kontinuierlicher Infusion über 48 h eine 26 % höhere Eliminationsrate bei Männern [41, 42]. Hierzu passend zeigten unterschiedliche Analysen eine klinisch relevante höhere Toxizität nach 5‑FU-Applikation bei Frauen mit vermehrtem Risiko von Grad-III- bis Grad-IV-Neutropenien, Nausea, Erbrechen und Diarrhöen [43]. Auch unter Kombinationschemotherapieregimen bei anderen Tumorentitäten zeigten sich für Frauen eine höhere Knochenmarkstoxizität sowie vermehrte gastrointestinale (GI) Nebenwirkungen [44, 45]. Neben dem vermehrten Auftreten von akuten Nebenwirkungen fand sich bei Frauen zudem eine höhere Rate an Spätfolgen wie der Kardiotoxizität nach anthrazyklinhaltiger Chemotherapie im Kindesalter [46, 47]. Frauen zeigen hier für jede kumulative Dosis einen höheren Verlust ihrer linksventrikulären Kontraktilität im Vergleich zu Männern, wobei der Geschlechtsunterschied mit steigender Kumulativdosis zunimmt [46, 47]. Eine Vielzahl weiterer in der Onkologie und Hämatologie verwendeter Therapeutika wird bei Männern schneller verstoffwechselt, einhergehend mit geringeren Serumkonzentrationen. Hierzu zählen neben Rituximab und 5‑FU auch Paclitaxel (20 % höhere Eliminationskapazität; [48]) und Bevacizumab (18–26 % schnellere Volumendistribution, 14 % höhere Clearance; [49]).

Zusammenfassend zeigen sich relevante geschlechtsspezifische Unterschiede in Pharmakologie und Toxikologie der onkologischen Therapieregime. Hieraus lassen sich Auswirkungen auf Therapieansprechen, Dosisdeeskalationen sowie die Lebensqualität der Patient*innen ableiten, denen im Zeitalter der personalisierten Medizin mehr Aufmerksamkeit geschenkt werden sollte. Klinische Studien zur Klärung, ob eine geschlechtsspezifische Dosierung von Chemo- und Immuntherapien Toxizität verringern und/oder Ansprechraten verbessern kann, sind vonnöten, um hieraus konkrete Anwendungen für den klinischen Alltag ableiten zu können.

## Gastrointestinale Tumoren

Insbesondere im Bereich der GI-Tumoren zeigen sowohl lange etablierte als auch aktuelle Studien eindrückliche Unterschiede zwischen den Geschlechtern: Eine große prospektive Kohortenstudie des National Cancer Institute Maryland zeigte, dass für Adenokarzinome des Ösophagus und Magenkarzinome der Kardia unter 21 untersuchten Krebserkrankungen die höchsten altersadjustierten Mann/Frau-Inzidenzratenverhältnisse („incidence rate ratios“ [IRR]) bestehen (Ösophaguskarzinome: IRR 12,19; 95 %-Konfidenzintervall [KI] 8,32–17,86; Magenkarzinome: IRR 4,93; 95 %-KI 3,59–6,77; [50]). Eine große aktuelle Datenanalyse aus 171 Krebsregistern in insgesamt 54 Ländern bestätigte den Trend mit etwas geringeren altersadjustierten Mann/Frau-Inzidenzratenverhältnissen („age-standardized incidence rate ratios“ [ASIR ratios]) von 6,7:1 für Ösophaguskarzinome und 4,0:1 für Kardiakarzinome [51].

Bei Magenkarzinomen besteht eine Übersterblichkeit unter jungen Patientinnen

Magenkarzinome liegen weiterhin unter den 10 häufigsten krebsassoziierten Todesursachen weltweit. Während die Inzidenz des Magenkarzinoms insgesamt in den letzten Jahrzehnten stetig abgenommen hat [52], verblieb der Anteil der (insbesondere jungen) Frauen hiervon großenteils unbeeinträchtigt [53]. Geschlechtsspezifische Unterschiede zeichnen sich bereits im konventionellen histopathologischen Subtyp ab. Siegelringzellige Adenokarzinome (SRC) betreffen häufiger Frauen und treten in jüngerem Alter auf, im Schnitt 7 Jahre vor Nicht-SRC [54]. Beachtet man, dass SRCs geringere Ansprechraten auf konventionelle Chemotherapeutika aufweisen, sich bei Diagnosestellung in fortgeschritteneren Stadien präsentieren und mit einem geringeren OS assoziiert sind [55, 56], werden die hohen klinischen Auswirkungen dieser geschlechtsspezifischen Inzidenz deutlich. Weitere Studien bestätigen, dass das weibliche Geschlecht mit den Faktoren junges Alter, diffuser Subtyp nach Laurén und SRC-Histologie assoziiert ist [56]. Während verschiedene Studien ein insgesamt schlechteres Outcome für Männer zeigen [57], besteht bei jüngeren Patent*innen (< 45 Jahren) eine weibliche Übersterblichkeit [56].

Magenkarzinome unterliegen einem eindrücklichen altersabhängigen Geschlechterverhältnis

Magenkarzinome unterliegen zudem einem eindrücklichen altersabhängigen Geschlechterverhältnis. Während um das 60. bis 65. Lebensjahr die größte Divergenz im Geschlechterverhältnis der Inzidenz besteht und der Männeranteil hier am höchsten ist, gleicht sich das Verhältnis jenseits davon (in beide Altersrichtungen) kontinuierlich an. Insbesondere mit jüngerem Patienten*innenalter zeigt sich ein zunehmender Frauenanteil mit einer Inversion des Geschlechterverhältnisses in der Altersgruppe unter 35 Jahren. Dies gilt insbesondere für Magenkarzinome jenseits der Kardia [51]. Ähnliche altersabhängige Geschlechtsverhältnisse („low-high-low male-to-female ratios“) werden auch bei anderen Tumorentitäten des GI-Trakts einschließlich Pankreas, Leber, Kolon und Rektum verzeichnet [51]. Dieses Muster zeigt sich für die genannten Tumoren einheitlich über 54 Länder und legt nahe, dass dies weniger auf Umwelt- und Expositionsfaktoren zurückzuführen ist, sondern auf biologischen Unterschieden zwischen den Geschlechtern beruhen könnte. Es besteht die Hypothese, dass Geschlechtshormone, insbesondere Östrogene, einen protektiven Einfluss hinsichtlich der Entwicklung von Neoplasien haben. Krebserkrankungen des GI-Trakts treten bei Frauen vermehrt in und nach der Menopause auf, die mit einem Östrogenabfall und damit Verlust des protektiven Faktors einhergeht [58, 59]. In Analysen der Singapore Chinese Health Study, einer großen prospektiven Kohortenstudie mit 34.022 eingeschlossenen Magenkarzinompatientinnen, stellten die Autor*innen dar, dass Reproduktionsfaktoren, die mit einem längeren Zeitfenster der Fruchtbarkeit verbunden sind, die Entwicklung von Magenkarzinomen verringern [60]. Hierzu zählen unter anderem ein höheres Alter bei natürlichem Beginn der Menopause (≥ 55 vs. < 45 Jahre: für mehrere Variablen bereinigte Hazard Ratio [HR] 0,50; 95 %-KI 0,25–0,99) sowie mehr Lebensjahre mit Menstruationszyklen (> 31,9 vs. ≤ 28,4 Jahre: HR 0,67; 95 %-KI 0,46–0,96). Darüber hinaus zeigte eine orale Kontrazeption (HR 0,67; 95 %-KI 0,47–0,94) oder eine orale Hormonersatztherapie für mehr als 3 Jahre (HR 0,40; 95 %-KI 0,17–0,90) eine Reduktion des Magenkarzinomrisikos [60, 61]. Weitere Studien unterstreichen diese Zusammenhänge [61–63], während bezüglich der klinischen Relevanz Kontroversen bestehen. Zudem scheint der beschriebene protektive Hormoneinfluss hauptsächlich für Nicht-SRCs vom intestinalen Typ zu bestehen. Eine Erklärung für den erhöhten weiblichen Anteil in jungem Diagnosealter und beim Siegelringzellsubtyp, der ein distinktes Expressionsmuster der Östrogenrezeptoren aufweist [29], ergibt sich hieraus ebenfalls nicht.

Eine neuere molekulare Charakterisierung von 295 primären Magenkarzinomen im Rahmen des The-Cancer-Genome-Atlas(TCGA)-Projekts definierte vier genomische Hauptsubtypen des Magenkarzinoms (Epstein-Barr-Virus[EBV]-infizierte, mikrosatelliteninstabile [MSI], genomisch stabile [GS] und chromosomal instabile [CIN] Tumoren) und konnte signifikante Unterschiede in der Geschlechterverteilung unter den genannten Subtypen feststellen [64]. MSI-Tumoren traten tendenziell häufiger bei weiblichen Patientinnen auf (56 %; *p* = 0,001), während die meisten EBV-positiven Tumoren von männlichen Patienten stammten (81 %; *p* = 0,037; [64]). Dass sich hieraus weitere geschlechtssensible Faktoren mit klinischen Auswirkungen ableiten lassen, zeigten Quaas et al. [65] in einer hochaktuellen, gepoolten Analyse von drei europäischen Kohorten. Hier zeigten Frauen in der Subgruppe der operablen MSI-high-Magenkarzinome einen signifikanten Überlebensvorteil im Vergleich zu ihrem männlichen Gegenpart. Zudem konnte gezeigt werden, dass ein MSI-high-Status nur für Patientinnen einen prognostisch günstigen Faktor darstellt.

Eine hohe Mikrosatelliteninstabilität ist nur für weibliche Patientinnen prognostisch günstig

Letztlich lässt sich festhalten, dass GI-Tumoren eine ausgeprägte geschlechtsdifferente Krankheitspräsentation aufweisen und weitere Untersuchungen auf hormoneller, genetischer und molekularer Ebene wünschenswert sind, um zugrunde liegende Mechanismen besser zu verstehen und geschlechtsspezifische präventive und therapeutische Angriffsstellen aufzudecken.

## Lymphome und Leukämien

Auch im Feld der Leukämien und Lymphome bestehen deutliche Unterschiede zwischen den Geschlechtern. Von einer chronischen lymphatischen Leukämie (CLL) als häufigster Leukämieform der westlichen Industrieländer im Erwachsenenalter sind Männer häufiger betroffen als Frauen. Die Deutsche CLL Studiengruppe (DCLLSG) zeigte 2017 in einer großen Metaanalyse mit 2247 Patient*innen, dass das weibliche Geschlecht ein unabhängiger Faktor für ein besseres Therapie-Outcome einer CLL-Erkrankung unter Erhalt von Rituximab-basierten Chemoimmuntherapien ist [12]. Patientinnen hatten ein verbessertes progressionsfreies Überleben („progression-free survival“ [PFS]) unter Therapie mit Fludarabin, Cyclophosphamid und Rituximab (FCR), Bendamustin und Rituximab (BR) sowie Chlorambucil und Rituximab (CLB-R) sowie ein längeres OS unter CLB-R-Therapie im Vergleich zu ihren männlichen Vergleichsgruppen [12].

Bei Männern weist Rituximab eine schnellere Clearance und einen geringeren Nutzen in der Kombinationstherapie auf

Vereinbar hiermit konnten pharmakokinetische Studien eine schnellere Clearance und damit geringe Exposition von Rituximab bei Männern zeigen [66]. Interessanterweise ließ sich kein weiblicher Vorteil im PFS oder OS unter Regimen mit anderen CD20-Antikörpern (beispielsweise Obinutuzumab) verzeichnen [12]. Ein anderer Erklärungsansatz für den weiblichen Überlebensvorteil ergibt sich aus den Auswertungen von vier (älteren) klinischen Studien (CLL1, 2 und 3 und LRF CLL4; [67, 68]) einschließlich 1821 bis dato unbehandelter CLL-Patient*innen. Frauen zeigten hier signifikant öfter prognostisch gute genetische Marker mit nichtmutiertem Immunoglobulin-heavy-chain-variable(*IGHV*)-Status (*p* = 0,007), fehlender *TP53*-Mutationen oder 11q-Deletion (*p* = 0,01) sowie CD38- (*p* = 0,0003) und *ZAP70*-Negativität (*p* = 0,05; [69]). Weiterführende Untersuchungen der geschlechtsspezifischen Unterschiede in Bezug auf das Therapieansprechen und die Verträglichkeit weiterer, aktuell die CLL-Behandlung bestimmender Therapeutika wie Bruton-Tyrosinkinase(BTK)-Inhibitoren und *BCL-2*-Inhibitoren fehlen derzeit, könnten aber spannende Erkenntnisse liefern.

Kohärent mit den Ergebnissen der DCLLSG zeigten mehrere Analysen von Patient*innendaten mit diffus großzelligem B‑Zell-Lymphom („diffuse large B‑cell lymphoma“ [DLBCL]), dass das männliche Geschlecht mit einem schlechteren Gesamtüberleben unter Rituximab-basierten Therapieregimen assoziiert ist [27]. Auch hier wird unter anderem die schnellere Rituximab-Clearance bei männlichen Patienten angeführt. Durch Einführung der Kombinationstherapie mit dem CD20-Antikörper und der bisherigen Standardtherapie mit Cyclophosphamid, Doxorubicin, Vincristin und Prednison in CHOP-21- und CHOP-14-Regimen konnte bekanntermaßen das Gesamtüberleben von DLBCL-Patient*innen signifikant verbessert werden [70]. Retrospektive Analysen konnten nun zeigen, dass die beiden Geschlechter in unterschiedlichem Ausmaß auf das angepasste Chemoimmuntherapieregim reagieren. Die Rate an kompletten Remissionen konnte unter Frauen von 68 % auf 83,7 % erhöht werden, während die Rate unter Männern von 63,9 % auf 76,6 % stieg [71]. Interessanterweise ziehen insbesondere ältere, männliche Patienten einen geringeren Nutzen aus der zusätzlichen Rituximabgabe im Vergleich zu jüngeren und/oder weiblichen Patient*innen. Auch hier werden unter anderem die schnellere Clearance, geringere Serumkonzentrationen und damit die kürzere Expositionszeit insbesondere in dieser Patientengruppe als mitursächlich vermutet [72, 73]. Pfreundschuh et al. [74] postulierten somit, dass Rituximab häufig in suboptimaler Dosierung verabreicht werde und zukünftige Studien zu optimalen, patientenindividuellen Dosen (geschlechts-, aber auch gewichts- und altersadaptiert) erfolgen sollten. Nicht zuletzt können sich aufgrund des breiten Einsatzes des CD20-Antikörpers hieraus auch Konsequenzen für weitere hämatologische und rheumatologische Krankheitsbilder ergeben.

Darüber hinaus konnte eine Analyse von Surveillance-Epidemiology-and-End-Results(SEER)-Medicare-Daten zeigen, dass der weibliche Überlebensvorteil sowohl in der Primär- als auch in der Salvage-Therapie besteht und unabhängig von der Behandlungsintention (aggressiv/kurativ vs. palliativ) ist. Zudem war das männliche Geschlecht hier ein Prädiktor für einen refraktären Verlauf bei Patient*innen mit DLBCL [75].

In einer hochaktuellen landesweiten epidemiologischen Kohortenstudie aus Schweden mit 20.738 Patient*innen (56,4 % männlich, 43,6 % weiblich) konnten für Männer signifikant höhere Inzidenzraten sowie eine Übersterblichkeit bei nahezu allen Lymphomsubtypen gezeigt werden [76], was die Ergebnisse vorheriger Studien einzelner Subtypen unterstreicht [77, 78]. Die Mann/Frau-IRR lagen zwischen 1,15 (95 %-KI 1,09–1,22) für follikuläre Lymphome und 5,95 (95 %-KI 4,89–7,24) für die Haarzellleukämie (HCL). Während sich lediglich bei den primär mediastinalen B‑Zell-Lymphomen (PMBL) ein signifikant höherer Frauenanteil zeigte (IRR 0,71), war der männliche Überanteil besonders ausgeprägt beim nodulären lymphozytenprädominanten Hodgkin-Lymphom (NLPHL; IRR 2,86), Burkitt-Lymphom (BL; IRR 2,92), Mantelzelllymphom (MCL; IRR 3,32) und der HCL. Zudem zeigte sich bei 13 von 16 Subtypen eine „male-to-female excess mortality rate“ > 1 (EMR, Übersterblichkeit), wobei diese letztlich nur für das klassische Hodgkin-Lymphom (EMR 1,26; 95 %-KI 1,04–1,54), aggressive nicht anderweitig klassifizierte Lymphome (EMR 1,29; 95 %-KI 1,08–1,55) und das kleine lymphozytische Lymphom („small lymphocytic lymphoma“ [SLL], EMR 1,52; 95 %-KI 1,11–2,07) signifikant blieb [76]. Auswertungen des dänischen Lymphomregisters (LYFO) erbrachten ähnliche Ergebnisse [19, 22]. Eine britische sowie eine weitere schwedische Studie legten darüber hinaus nahe, dass das männliche Geschlecht bei Patienten mit DLBCL (oder follikulärem Lymphom) auch nach Bereinigung für mögliche Kofaktoren wie Komorbidität, sozioökonomischen Status sowie lymphombezogene Risikofaktoren ein unabhängiger negativer prognostischer Faktor bleibt [23, 24]. Bis auf wenige Ausnahmen wie den International Prognostic Score (IPS) zur Risikostratifizierung des Hodgkin-Lymphoms [79] findet das Geschlecht als prognostischer Faktor in klinisch relevanten Lymphomscores kaum Beachtung. Zudem sind die Ursachen und Mechanismen für die lymphomsubtypenübergreifenden geschlechtsspezifischen Unterschiede in Inzidenz und Übersterblichkeit größtenteils weiterhin ungeklärt. Ein besseres molekulares Verständnis könnte die patientenindividuelle Therapie vorantreiben und das Wissen über Ätiologie und Biologie der B‑Zell-Lymphome erweitern.

## Lungenkarzinome

Lungenkarzinome stellen ein weiteres imposantes Beispiel der Onkologie für unterschiedliche Einflüsse des Geschlechts auf Krankheitsinzidenz, Histologie und Therapieansprechen dar.

Während die Gesamtinzidenz von Lungenkarzinomen in den letzten Jahrzehnten insgesamt erfreulicherweise gesunken ist, hat sich das ehemals deutlich höhere Mann/Frau-Verhältnis zunehmend angeglichen. Ferner zeigt sich heute ein signifikant höherer Frauenanteil in jüngeren Altersgruppen (30–49 Jahre; [80]). Histopathologisch präsentieren Frauen im Vergleich zu Männern häufiger Adenokarzinome [81]. Insbesondere der Anteil der Adenokarzinome unter jungen (nicht rauchenden) Patientinnen stieg innerhalb der letzten Jahre an [82]. Auch wenn Tabakkonsum weiterhin den führenden Risikofaktor für die Entwicklung von Karzinomen der Lunge darstellt und sich das Rauchverhalten geschlechtsspezifisch in ähnlicher Tendenz verändert hat [83], gilt dies hauptsächlich für Plattenepithelkarzinome, und verhaltensbezogene Erklärungen sind nicht ausreichend für den zunehmenden Anteil junger Frauen. Auf biologischer Ebene wurden unter anderem bei rauchenden Frauen höhere CYP1A1-Level gefunden (signifikant korrelierend mit DNA-Addukten der Lunge), die mit einer höheren Anfälligkeit für die Entwicklung von Lungenkarzinomen assoziiert sind [84]. Auch in anderen Fachdisziplinen bestehen unter Tabakkonsum erhöhte Gesundheitsrisiken für Frauen, so birgt der regelmäßige Konsum ein 35 % höheres Risiko für die Entwicklung kardiovaskulärer Erkrankungen gegenüber Männern [85]. Allerdings liegen zur Hypothese, dass Frauen auf Tabaknoxen sensibler mit der Entwicklung von Karzinomen reagieren, kontroverse Daten vor [86, 87].

Im Hinblick auf das geschlechtsspezifische Outcome bei Patient*innen mit nichtkleinzelligem Lungenkarzinom („non-small-cell lung cancer“ [NSCLC]) zeigen viele Studien aus unterschiedlichen geografischen Regionen übereinstimmende Ergebnisse: Männer weisen im Vergleich zu Frauen eine insgesamt schlechtere Prognose auf [88–90]. Eine gepoolte Analyse von fünf randomisierten Phase-III-Studien bei fortgeschrittenen NSCLC mit 2349 Patient*innen bestätigte den weiblichen prognostischen Vorteil unter platinbasierten Chemotherapien (HR 0,83; 95 %-KI 0,74–0,92; *p* = 0,0005), wobei ein längeres Gesamtüberleben in näherer Analyse nur auf den histologischen Subtyp der Adenokarzinome beschränkt zu sein scheint [91]. Eine prospektive Studie untersuchte zudem geschlechtsspezifische Risikofaktoren bei Patient*innen mit NSCLC im Stadium I–III. Hier zeigten Frauen ein signifikant besseres 5‑Jahres-Überleben für jedes Stadium (Stadium I: 69 % vs. 64 %; Stadium II: 60 % vs. 50 %; Stadium III: 46 % vs. 37 %). Sie präsentierten frühere Stadien, waren bei Diagnose jünger und konnten häufiger eine partielle oder komplette Remission unter neoadjuvanter Chemotherapie erreichen [81].

Frauen profitieren mehr von EGFR-Inhibitoren als Männer

Heute ist die Behandlung der Lungenkarzinome einer der führenden onkologischen Bereiche der Immun- und zielgerichteten Therapien. Geschlechtsverschiedene Ansprechraten sind hier zwischen den unterschiedlichen Targets besonders ausgeprägt. Unter anderem zeigen weibliche Patientinnen mit NSCLC in einer Metaanalyse von sechs Phase-III-Studien einen höheren Nutzen unter Therapie mit Epidermal-growth-factor-receptor(EGFR)-Inhibitoren (Gefitinib, Erlotinib oder Afatinib) im Vergleich zu konventioneller Chemotherapie (HR 0,34; 95 %-KI 0,28–0,40 für Frauen vs. HR 0,44; 95 %-KI 0,34–0,56 für Männer; *p* < 0,0001; [92]). EGFR-Inhibitoren wurden hier in der Erstlinientherapie eingesetzt und es wurden nur Patient*innen mit nachgewiesen sensiblen EGFR-Mutationen eingeschlossen [92]. Bei anderen EGFR-Inhibitoren, wie dem monoklonalen Antikörper Cetuximab, waren hingegen keine Unterschiede zwischen den Geschlechtern zu verzeichnen [93]. Die Prävalenz von EGFR-Mutationen sowie ihre mRNA-Expression bei Patient*innen mit NSCLC ist unter Frauen insgesamt deutlich höher als unter Männern (43,7 % vs. 24,0 %; [94, 95]) und kann somit zu einer besseren Ansprechrate unter Frauen beitragen. Valide geschlechtssensible Daten zu Ansprechraten neuerer Tyrosinkinaseinhibitoren (dritte Generation) sind somit von großem Interesse und sollten zukünftig erhoben werden.

Anders scheint es sich bei Antikörpern gegen das „programmed cell death protein 1“ (PD-1) zu verhalten. In einer großen Metaanalyse wurden zwei Studien zum Vergleich einer Pembrolizumab-Monotherapie mit einer Chemotherapie (KEYNOTE-010 und KEYNOTE-024 [96, 97]) sowie drei Phase-III-Studien zum Vergleich einer Nivolumab-Monotherapie mit einer konventionellen Chemotherapie (CheckMate 017, CheckMate 026, CheckMate 057 [97–99]) für fortgeschrittene NSCLC im Hinblick auf geschlechtsspezifische Unterschiede im Outcome untersucht [92]. Es konnte ein deutlicher Nutzen unter Anti-PD-1-Antikörper-Therapie für männliche Patienten gezeigt werden (HR 0,76), wobei sich deutliche Schwankungen zwischen den Studien zeigten [92]. Insgesamt ermittelte die Metaanalyse für Männer eine Reduktion des Progressionsrisikos um 24 % unter Anti-PD-1-Antikörper-Therapie. Bei weiblichen Patientinnen war hingegen kein signifikanter Nutzen einer Nivolumab- oder Pembrolizumab-Monotherapie gegenüber den konventionellen Chemotherapien zu verzeichnen (HR 1,03; 95 %-KI 0,89–1,03; *p* = 0,69; [92]).

Insgesamt lässt sich feststellen, dass sich die Prognose von NSCLC-Patient*innen deutlich zwischen Männern und Frauen unterscheidet. Je nach Therapie und Target zeigen sich hier teils signifikante Nutzen entweder für das männliche oder für das weibliche Geschlecht. Erstaunlicherweise fand sich ein besseres Ansprechen bei Frauen eher unter (teils veralteten) Chemotherapieregimen, während Männer mehr von neueren Immun- und zielgerichteten Therapien zu profitieren scheinen. Weitere detaillierte Übersichten hierzu sind jedoch erforderlich.

### Weitere Informationen zum Thema

- https://www.esmo.org/about-esmo/organisational-structure/esmo-task-forces/esmo-gender-medicine-task-force
- https://medfak.uni-koeln.de/forschung/forschung-an-der-fakultaet-1/ag-sex-gender-and-diversity-in-medical-research

## Generelles und Schlussfolgerung

Es ist noch gar nicht lange her, dass die National Institutes of Health (NIH) der USA erstmalig festlegten, dass in von ihnen geförderten Forschungsprojekten das Geschlecht als biologische Variable berücksichtigt werden muss [100]. Trotz deutlichem Fortschritt in den letzten Jahren sind Frauen auch heute noch in klinischen Studien (inklusive Phase-III-Studien) unterrepräsentiert [101]. Eine amerikanische Übersichtsarbeit zeigte, dass nur 37 % der in Phase-III-Studien eingeschlossenen Patient*innen weiblich sind und eine Mehrzahl der Studien (64 %) keine geschlechtsspezifischen Ergebnisse liefert [102]. Nimmt man das Geschlecht als eine relevante biologische Variable ernst, ist dies eine Tatsache, die man im wissenschaftlichen Kontext kaum akzeptieren kann.

Neben dem zunehmenden Bewusstsein für die hier diskutierten geschlechtsspezifischen Unterschiede könnten zukünftig weitere patientenindividuelle Faktoren wie Gender und Ethnizität eine wachsende Rolle für eine optimale Patient*innenversorgung spielen und sollten in Studien adressiert werden.

Gemäß den Schlussfolgerungen des Workshops „Gender medicine and oncology“ der European Society for Medical Oncology [103] sollten Frauen und Männer mit Tumorerkrankungen mit signifikanten Unterschieden in Epidemiologie und Prognose als biologisch differente Gruppen angesehen werden und individuelle Therapieansätze untersucht werden. Während bei einer Vielzahl der untersuchten Krebserkrankungen eine erhöhte Inzidenz und Übersterblichkeit für Männer besteht, konnten wir zeigen, dass dies zwischen den Erkrankungen schwanken kann und insbesondere die Untergruppe der jungen Frauen in der Onkologie häufig eine Sonderstellung einnimmt, der bislang nur wenig Aufmerksamkeit geschenkt wurde.

Abschließend ist festzuhalten, dass viele relevante Unterschiede im Outcome verschiedenster Tumorerkrankungen bestehen und die Berücksichtigung und Erforschung zugrunde liegender biologischer Aspekte und klinischer Einflüsse Bestandteil zukünftiger Studien sein sollten. Dies ist ein Plädoyer für eine geschlechtssensible Wissenschaft und für deren Implementierung in den klinischen Alltag, insbesondere in einem so stark patientenindividualisierten Fach wie der Hämatologie und Onkologie.

## Fazit für die Praxis

- Das Geschlecht ist ein relevanter prognostischer Faktor bei einer Vielzahl von Krebserkrankungen.
- Geschlechtsspezifische Unterschiede bestehen unter anderem in Immunologie, Hormonhaushalt und Pharmakologie und nehmen signifikanten Einfluss auf die onkologische Behandlung und deren Ansprechraten.
- Eine fortschrittliche patientenindividualisierte onkologische Therapie wird sich daran messen müssen, die bestehenden relevanten Unterschiede zwischen den Geschlechtern im klinischen Alltag zu berücksichtigen und in zukünftigen Studien zugrunde liegende Mechanismen zu adressieren, um eine bestmögliche Behandlung für Frauen und Männer in der Onkologie garantieren zu können.
